# Young people are medically invulnerable to COVID-19 but vulnerable in the labor market: Korean evidence

**DOI:** 10.1186/s13561-022-00360-4

**Published:** 2022-02-22

**Authors:** Saejung Park, Joonmo Cho

**Affiliations:** School of Economics, Sungkyunkwan University, Postal address: 32413, 53 Myeongryun-Dong 3-ga, Jongno-gu, Seoul, 110-745 South Korea

**Keywords:** COVID-19, Discouraged worker, Economically inactive population, Unemployment, Youth unemployment

## Abstract

**Background:**

COVID-19 and its preventive measures affect not only the state of public health but also the economy. The economic impact of COVID-19 varies depending on age, and it is argued that young people have experienced the greatest negative impacts.

**Methods:**

This study was an analysis of the impact of the COVID-19 outbreak in January 2020 on the Korean labor market. Large-scale data from the Economically Active Population Survey from January to June of 2018 to 2020 were used when analyzing the impacts of COVID-19 on unemployment and the economically inactive population by age group. Through this study’s empirical analysis, we examined for this study whether the COVID-19 outbreak has affected the labor market differently based on age. By analyzing the interaction terms of the young person’s status and the time of the COVID-19 outbreak, we determined the impacts of the outbreak on economic inactivity among young people, as well as examining the reasons for these differential effects.

**Results:**

Compared with the middle-aged or older group, young people were more likely to become economically inactive than unemployed. Our empirical results using multinomial logistic regression revealed several reasons for the increase in economic inactivity, such as discouragement during the job search, childcare, housework, and studying at an institution, along with other determinants of economic inactivity. Young people showed a significantly higher relative probability of becoming economically inactive or discouraged job seekers following the COVID-19 outbreak when compared to other age groups. In addition, through the analysis of the possibility of employment, the young people responded negatively to the possibility of employment in the future compared to the middle-aged after COVID-19.

**Conclusion:**

Young people in South Korea possess little career experience in the labor market and tend to be seeking work rather than working. Because economic activities are likely to shrink structurally during a pandemic, it is necessary to empirically determine the damage incurred by people who are vulnerable in the labor markets, such as the younger population which was the subject of this study. Accordingly, future policy directions are suggested for the prevention of a rapid increase in the rate of economic inactivity among the younger population during the pandemic.

## Introduction

In order to inform the younger population of the dangers of SARS-CoV-2 virus (COVID-19), the World Health Organization’s (WHO) Secretary-General said in March 2020, “Young people are not invincible. The elderly has been hit hardest by COVID-19, but young people won’t be spared, either [1].” Amidst a worldwide public health emergency, many people are being infected by the virus regardless of age, and young people are no exception. Korea witnessed the first confirmed case of COVID-19 on January 20, 2020, and the cumulative number of confirmed cases as of July 31 measured at 14,305 (local outbreaks: 11,909; imported cases: 2396) [2]. The highest number of confirmed cases was recorded within the 20–29 age group. However, none of the Korean cases under age 30 died, and nearly all of the deceased, at a rate of 93%, were age 60 or above.

With COVID-19 tracking, Korea is transforming its disease control system while consistently emphasizing social distancing to avoid contact with other members of society [3]. Social distancing is a disease control mechanism that inhibits face-to-face contact with others to an extent that is so intense that it affects economic activity [4]. It is essential however as the COVID-19 coronavirus is able to transfer from the host (person) to other individuals through the transmission of respiratory droplets [5]. In addition, some cases are asymptomatic, with 40% of the confirmed cases in Vo, Italy, in one study being asymptomatic [6]. In another study, 44% of cases of secondary transmission were caused by transmission from asymptomatic patients [7]. Initially, in Korea, there were many younger individuals who were asymptomatic or showed mild symptoms; however, they were medically invulnerable and went about their daily activities without knowing that they were infected. As a result of this, as well as the younger individuals’ wider range of activities when compared to that of older people, these younger individuals were more likely to infect others [2].

COVID-19 and its preventive measures affect not only the state of public health but also the economy. For instance, recent researchers established that the U.S. employment rate fell following the onset of the COVID-19 pandemic, and that the unemployment rate and the economically inactive population increased significantly [8]. A labor market as flexible as that of the United States has an unemployment rate which responds sensitively to economic circumstances, and negative economic events may cause the economically inactive population to increase along with unemployed population [9], though this relationship does not also hold for every country. Quantitative shifts between unemployment and economically inactive populations may vary depending on the labor market structure, social safety net, and government COVID-19 policies.

The economic impact of COVID-19 varies depending on age, and it is argued that young people have experienced the greatest negative impacts. The International Labour Organization (ILO) (2020) found that COVID-19 has had a catastrophic and disproportionate impact on adolescent employment worldwide. The United Nations observed that people below the age of 25 now face a higher likelihood of unemployment, discontinued education and training, and greater additional barriers when entering the labor market due to COVID-19, which could result in the emergence of a “lockdown generation” [10]. Recently in Korea, firms postponed the new recruitment of permanent or regular employees due to COVID-19, which markedly decreased the number of permanent employment opportunities for young people [11]. Previous studies have shown that natural disasters such as pandemics do not affect everyone equally [12, 13]. In fact, their unequal spread of their effects can in fact aggravate pre-existing inequalities [14].

Young people usually possess little professional experience when they leave school to search for work or pursue vocational training and internships [15]. The reduced level of economic activity due to COVID-19 resulted in the interruption of young people’s efforts to enter labor markets, along with many other professional pursuits. Therefore, they are now experiencing the full brunt of the wide macroeconomic damage caused by COVID-19, and are currently the most vulnerable population in the labor market.

Through this study’s empirical analysis, we examined for this study whether the COVID-19 outbreak has affected the labor market differently based on age. Specifically, we examined the changes in the employment rate, unemployment rate, and economically inactive population before and after COVID-19, with a focus on young people aged between 15 and 29 years. By analyzing the interaction terms of the young person’s status and the time of the COVID-19 outbreak, we determined the impacts of the outbreak on economic inactivity among young people, as well as examining the reasons for these differential effects.

Therefore, this study is structured as follows. Chapter 2 introduces the COVID-19 situation based on confirmed cases in Korea and the Korean government’s subsequent quarantine measures. Chapter 3 summarizes related prior studies. Chapter 4 explains the Economically Active Population Survey which was used for this analysis, while also explaining the basic statistical analysis and its methods. Chapter 5 discusses the analytical results of the effects of COVID-19 on economic activity by age group. Chapter 6 recaps the results and presents the implications of the study, and the future policy suggestions for the government.

### COVID-19 in Korea

COVID-19 was first discovered in Wuhan, China, in December 2019. It has since spread rapidly worldwide [16]. Following the first confirmed case in Korea on January 20, 2020, the Korean government raised the crisis alert level from blue to yellow and set up the Central Disease Control Headquarters (KCDC). As the number of confirmed cases increased to four on January 27, the government raised the level to orange, and thereafter, the case of Patient 31 occurred, who was an individual who had participated in religious activities in Daegu, a city in southeast Korea. Patient 31 infected an extremely high number of participants during the course of these religious activities, and the government raised the crisis alert level to red, the highest; while the command center was also moved from the KCDC to the Central Disaster and Safety Countermeasures Headquarters, with the crisis alert level in Korea remaining red until July 2020 [3].

**Fig. 1:**
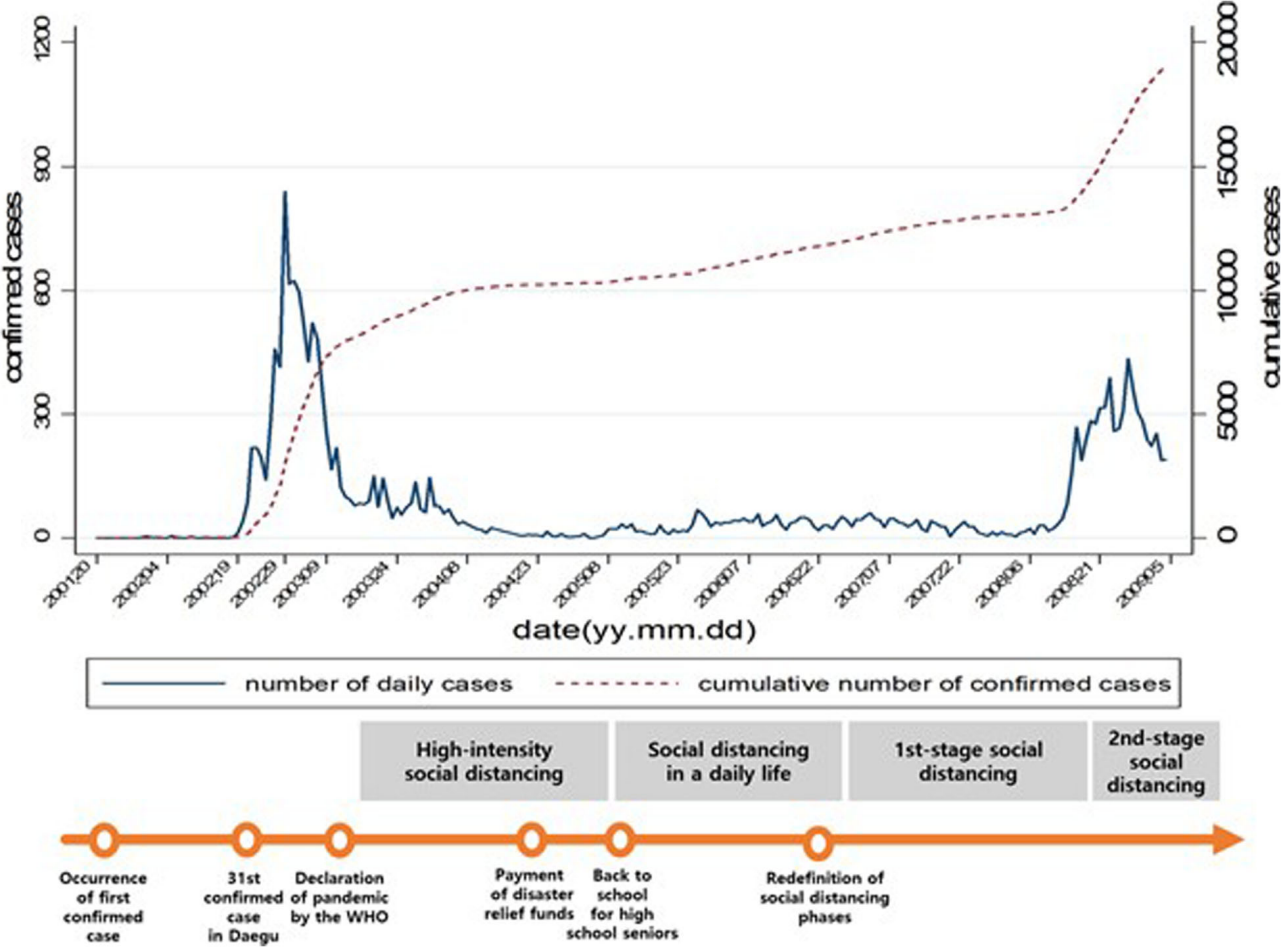
Trends in the number of confirmed COVID-19 cases in Korea, quarantine measures, and important events

Figure 1 shows the trend in the daily number of confirmed COVID-19 cases and the cumulative number of confirmed cases in Korea. The solid line in the graph represents the number of confirmed cases per day, and is interpreted using the Y-axis on the left. The dotted line represents the cumulative number of confirmed cases and is interpreted using the Y-axis on the right. The X-axis represents the date, with the first two digits representing the year, the third and fourth numbers representing the month, and the last two digits representing the date. In addition, it shows the social distancing phases implemented by the Korean government in line with the trend in the number of confirmed cases, as well as the timeline of important events which occurred in Korea at the same time. It can be seen in Fig. 1 that both the number of confirmed cases and the cumulative number of confirmed cases have increased rapidly from February, when the 31st patient was diagnosed in Daegu. The number of confirmed cases per day peaked at 813 individuals on February 29, with the cumulative number exceeding 3000 individuals. As the number of COVID-19 confirmed cases soared, the government realized the severity of the disease’s spread, and decided that contact between people should be cut off in order to prevent the further spread of the disease, enforcing a minimum level of social distancing between people in order to prevent transmission through respiratory droplets. The government established an everyday state referred to as “social distancing” and launched a campaign to minimize contact between people in most public and private spaces. Thereafter, the government implemented social distancing in phases, implementing Phase 1 when the number of confirmed cases is relatively low, and leveling up all the way to Phase 5 when the number of confirmed cases becomes high when taking into consideration the number of confirmed cases and economic conditions. Thus far, the government enacted social distancing up until Phase 2.5. Furthermore, high school seniors who had to take the college entrance exam later in the year first began going to school, to be followed sequentially by students of lower grades, as per the social distancing implementation measures. The Korean central and local governments also provided emergency disaster support funds of approximately 100,000 won per person within the general public, in order to alleviate the difficult economic situation most people had been experiencing due to the pandemic.

As of July 31, there were 14,305 confirmed cases in Korea, 2.76 confirmed cases per million people, with 301 fatalities, and a fatality rate of 2.10%. The government’s preventive measures included rapid diagnosis and tracing, health care workers’ volunteering, and the public’s participation in social distancing, with all of this leading to a rapid decline in the number of confirmed cases by mid-March. Internationally and domestically, Korea was commended for successfully battling the disease without the enforcement of massive lockdown or curfew [17].

Korea undertook various measures to control and prevent the spread of COVID-19 by improving the national defense system in order to correct vulnerabilities revealed during the response to MERS in 2015 [18]. The primary measures were to develop a real-time PCR protocol that could give diagnoses in six hours, and then to test anyone who came in contact with an infected individual or visited high-risk locations [3]. Beginning with the first confirmed case, the government disclosed detailed results of epidemiological investigations on its website. Individuals with symptoms or who had come in contact with an infected individual were to self-quarantine to prevent infecting others, and a smart phone application was developed to monitor the individuals in quarantine.

**Fig. 2:**
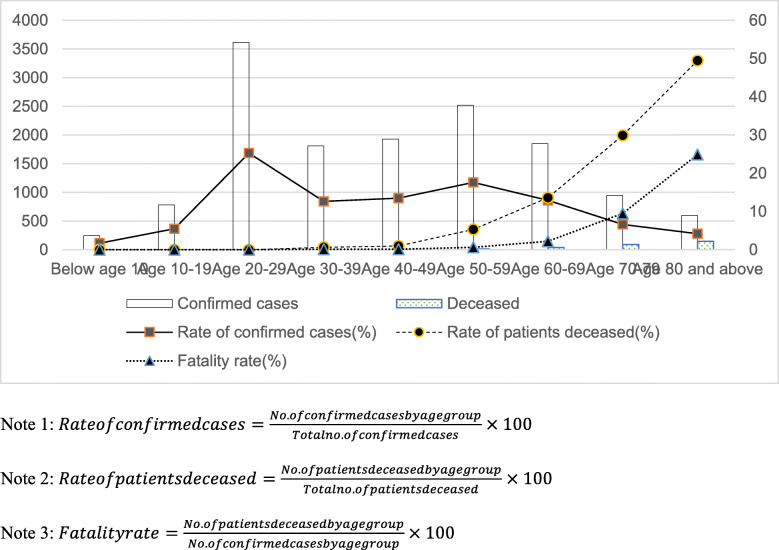
Confirmed cases of COVID-19 by age group (as of 12:00 a.m. on July 31, 2020)

Figure 2 shows the current number of confirmed cases in Korea by age, and the bars with no patterns represent the number of confirmed cases by age group. The highest number of confirmed cases, 3620, occurred among Koreans in their 20s, accounting for 25.31% of all confirmed cases; however, despite these numbers, their fatality rate was 0%, and the fatality rate actually increased by age group: 50s, 5.32%; 60s, 13.62%; 70s, 29.90%; 80s, 49.50% [19]. In addition to identifying the highest infection rate among residents in their 20s, the fatality rate was highest among those in their 80s.

### Relevant literature

The number of confirmed cases of COVID-19 has increased rapidly around the world since its outbreak. WHO declared the COVID-19 outbreak a pandemic on March 9, due to both the rate of its rapid spread and its higher mortality rate.

Numerous studies on COVID-19 have been published since its outbreak, as this unexpected “natural disaster” affects not only the state of public health, but also the economy and the labor market of many countries. Various studies have been conducted, including economic research papers confirming the impact of the COVID-19 outbreak on macroeconomic indicators such as GDP, oil prices, and stock prices, studies on the impacts on the labor market such as employment, unemployment, and inequality, and studies on the impacts on the environment such as changes in air pollution.

Of these, there have been a series of studies specifically focusing on how the impact of COVID-19 has impacted individuals’ economic circumstances differently, depending on their income bracket and industry. A representative study by Chetty et al. (2020) showed that there was a significant difference in the duration and size of employment loss based on groups’ income classes following the COVID-19 outbreak [20]. In particular, the percent change in employment rates of the high-income class showed a lower decline than that of the low-income class. In addition, the recovery in employment was faster for those in the high-income class. Chetty et al. (2020) also confirmed spending changes by category following the COVID-19 outbreak [20]. Spending in businesses such as beauty parlors, air transport-related businesses, and food services, showed a sharp decline in April 2020, compared to February 2020. Spending in the beauty parlor industry, in particular, plunged by about 100%. Overall, declines in spending depending by industry can be widely observed.

Jaimovich and Siu (2020) confirmed the recession’s significant adverse impact on the income of wage workers by income class, which declined overall as a result of lower corporate profits after the COVID-19 outbreak [21]. It was also shown that while the employment rate of those in the bottom 25% percentile of the wage distribution declined 34% in April 2020 when compared to before the COVID-19 outbreak, the employment rate of those in the top 25% percentile decreased only 10%. Furthermore, the employment rate for the top 25% percentile showed a rebound to their previous levels by the end of May, but the employment rate for those in the bottom 25% percentile continued to show a 17% decline in September 2020, compared to before the COVID-19 outbreak. In other words, Jaimovich and Siu (2020) showed that job losses were greater and lasted longer for low-wage workers [21].

A representative study of the impact of the COVID-19 spread on employment in Korea is that of H. Jung (2020) [22]. Jung used the employment insurance database and Worknet data to confirm the changes in the number of unemployment benefit applications and new job openings following the COVID-19 outbreak, focusing on differences in gender and region. It was confirmed that the number of employment insurance holders in Korea decreased by about 20% during the March–May 2020 period, compared to before the COVID-19 outbreak, and that the decline was greater for women than for men. In addition, the number of job openings on Worknet declined significantly in Seoul and in the South Jeolla Province compared to other regions, showing that the impact of COVID-19 on employment was felt differently across regions in Korea.

J. Lee (2020) analyzed the effects of COVID-19 on jobs in specific industries using the synthetic control method [23]. Specifically, his study showed that the demand for local service industries, such as healthcare, education, and travel, which are not essential to life, declined sharply due to the risk of contracting COVID-19, resulting in a significant decrease in jobs in these industries. In particular, the expected counterfactual of the number of employed workers in the case that COVID-19 had not occurred was estimated using the synthetic control method, by combining the trend in the actual number of employed workers before the COVID-19 outbreak with past similar trends of employed workers. As a result, it was estimated that about 1.08 million jobs in those industries were lost in April 2020.

Similar studies analyzing the effects of natural disasters revolving around infectious diseases, which had been conducted before the COVID-19 outbreak, showed that the effects can differ depending on the industry and the class. Lee and Warner (2005) showed that consumer spending in service industries requiring face-to-face contact decreased sharply in Hong Kong due to the SARS outbreak of 2003, which led to salary cuts, unemployment, workplace closures, and severe damage to service workers based on their qualitative research [24]. Furthermore, Lee and Cho (2016) showed that the spread of MERS of 2015 in Korea adversely affected the employment of the elderly, who are particularly vulnerable within the labor market [25].

Natural disasters do not affect everyone equally - not only in terms of public health but also in terms of economics – and in fact, they affect people differently depending on a variety of different characteristics. Therefore, this study focuses on the impact of the spread of COVID-19 on the labor market by age group in Korea. The reason for choosing to focus on young people in particular, is due to the stylized fact that the Korean labor market for young people has a particularly weak structure that is very sensitive to economic fluctuations and has a tendency for overshooting during market changes [26]. In addition, it is highly likely that young people will become unemployed, falling into the state of being out of work or becoming a member of the economically inactive population if the transition from school to the labor market is not smooth due to complications which arose from COVID-19. Young people are suffering from the postponement and reduction of new, large-scale hiring announcements by companies. The reason for focusing on the impacts on the labor market is because, according to Shama and Krishna (2007), current employment levels and unemployment are known to affect the living standards, disposable income, education, savings, and the psyche of individuals [27]. If it can be found that the COVID-19 outbreak has a greater negative impact on certain groups, such vulnerable groups can be identified in the future and tailored policy support can be provided for them.

## Methods

### Data

Analyzing any impacts on labor markets requires data on economic markers such as employment and unemployment rates and labor characteristics. The Economically Active Population Survey (EAPS) by Statistics Korea is a survey that determines the monthly supply of labor, and the survey data inform the government’s monthly reported employment and unemployment rates. The EAPS was suitable for analyzing the impact of COVID-19 on the labor market because it reveals monthly changes in economic conditions.

We analyzed COVID-19’s impact on employment using the EAPS data; specifically, the subjects of analysis included people aged 15–70 years who were unemployed or economically inactive during the disease outbreak. Data from January to June 2018, 2019, and 2020 were included, with the period from January to June 2020 typified as the post period. During this period of analysis, a total of 331,028 people were sampled. Of these, 23,229 were unemployed and 307,799 were economically inactive. This study did not require approval from the institutional review board because the EAPS dataset consists of secondary data that do not include personal information.

Table 1 shows the distribution of sample population according to age and the state of economic activity. The proportion of young people who were economically inactive was larger than that of middle-aged people. However, the proportion of inactive young people decreased slightly in 2019, but again increased in 2020. The proportion of the elderly economically inactive population steadily increased from 2018 to 2020.

**Table 1:** Sample distribution according to age and the state of economic activity (Unit: person)

Year	The state of economic activity	Young(15–29)	Middle-aged (30–49)	Elderly (50 over)
2018	Unemployed	2598	2549	2387
	Economically inactive	34,939	24,544	44,636
2019	Unemployed	2594	2540	2783
	Economically inactive	33,648	23,681	44,793
2020	Unemployed	2324	2381	3073
	Economically inactive	33,793	22,754	45,011

This study analyzed three things. First, it investigated the state of economic activity by age group before and after COVID −19. To analyze the economic activity state, a variable with a value of 1 was assigned as a dependent variable if a person’s state is economically inactive and 0 if unemployed. This study investigates how the economic activity status has changed by age group before and after COVID − 19 using this variable. The second dependent variable was the reason for not looking for a job. The variable divides the reasons for not looking for a job into four main categories; “discouraged about job search,” “childcare, housework, and studying at an institution,” “mental/physical disabilities, others and non-response,” and “unemployment.” Third, the possibility of employment was analyzed. In response to this variable, individuals responded to the question on whether they could get a job on their own. This variable investigates whether young people perceive the employment situation as more negative than middle-aged people after the outbreak of COVID − 19.

Table 2 shows the proportion of samples that are economically inactive, by the reason for economic inactivity. In other words, Table 2 shows the reasons for economic inactivity by year for the entire sample excluding non-responses, and the reasons for economic inactivity by year for young people aged 15–29. An increasing number of young people aged 15–29 gave up their job search in 2020 following the COVID-19 outbreak, due to reasons such as “No job that correctly fits my major or career experience,” “No job with the wage level or working conditions I want,” and “No job nearby,” all of which demonstrate that while young people want to work and have the necessary skills for the jobs, they do not have the years of work experience required by the labor market. Meanwhile, all samples showed an increase of individuals falling into an economically inactive state, with reasons such as “No job nearby,” “Studying in school” and “Others” in 2020.

**Table 2:** Distribution of sample according to the reasons for economic inactivity

	Age	Young (15–29)			All samples		
	Year	2018	2019	2020	2018	2019	2020
Reason for being a discouraged worker	No job that correctly fits my major or career experience	0.14	0.13	0.15	0.10	0.10	0.10
	No job with the wage level or working conditions I want	0.25	0.25	0.26	0.24	0.25	0.24
	No job nearby	0.02	0.03	0.04	0.11	0.11	0.12
	Lack of education, skills, experience	0.41	0.41	0.33	0.18	0.18	0.16
	The employer may think I am too young or too old	0.00	0.00	0.00	0.08	0.07	0.05
	There was no job when I searched earlier	0.07	0.06	0.06	0.16	0.17	0.17
Housework	Childcare	0.00	0.00	0.01	0.02	0.01	0.02
	Housework	0.00	0.00	0.00	0.04	0.04	0.04
	Studying at an institution	0.03	0.04	0.04	0.01	0.01	0.02
Others	Mental and physical disorder	0.00	0.00	0.00	0.00	0.00	0.00
	Others	0.07	0.08	0.10	0.06	0.06	0.08
raw sample size		3907	4180	4784	13,083	13,099	14,661

Table 3 shows the sample distribution according to the possibility of employment. There were 36,958 respondents. More than 90% of the respondents were positive employment.

**Table 3:** Sample distribution according to employability

Age group	Possibility		No possibility		Total
	*N*	%	*N*	%	
young	11,850	0.92	1021	0.08	12,871
middle-aged	9584	0.91	945	0.09	10,529
the elderly	12,699	0.94	859	0.06	13,558

### Basic statistics

The rampant nature of the coronavirus infection led the Korean government to advise people to avoid leaving their homes during the most severe parts of the pandemic. This, along with social distancing, significantly affected the Korean economy over the last seven months. Despite the OECD’s previous positive predictions for the Korean economy, the critical economic effects of COVID-19 included a declining employment rate in Korea.

**Fig. 3:**
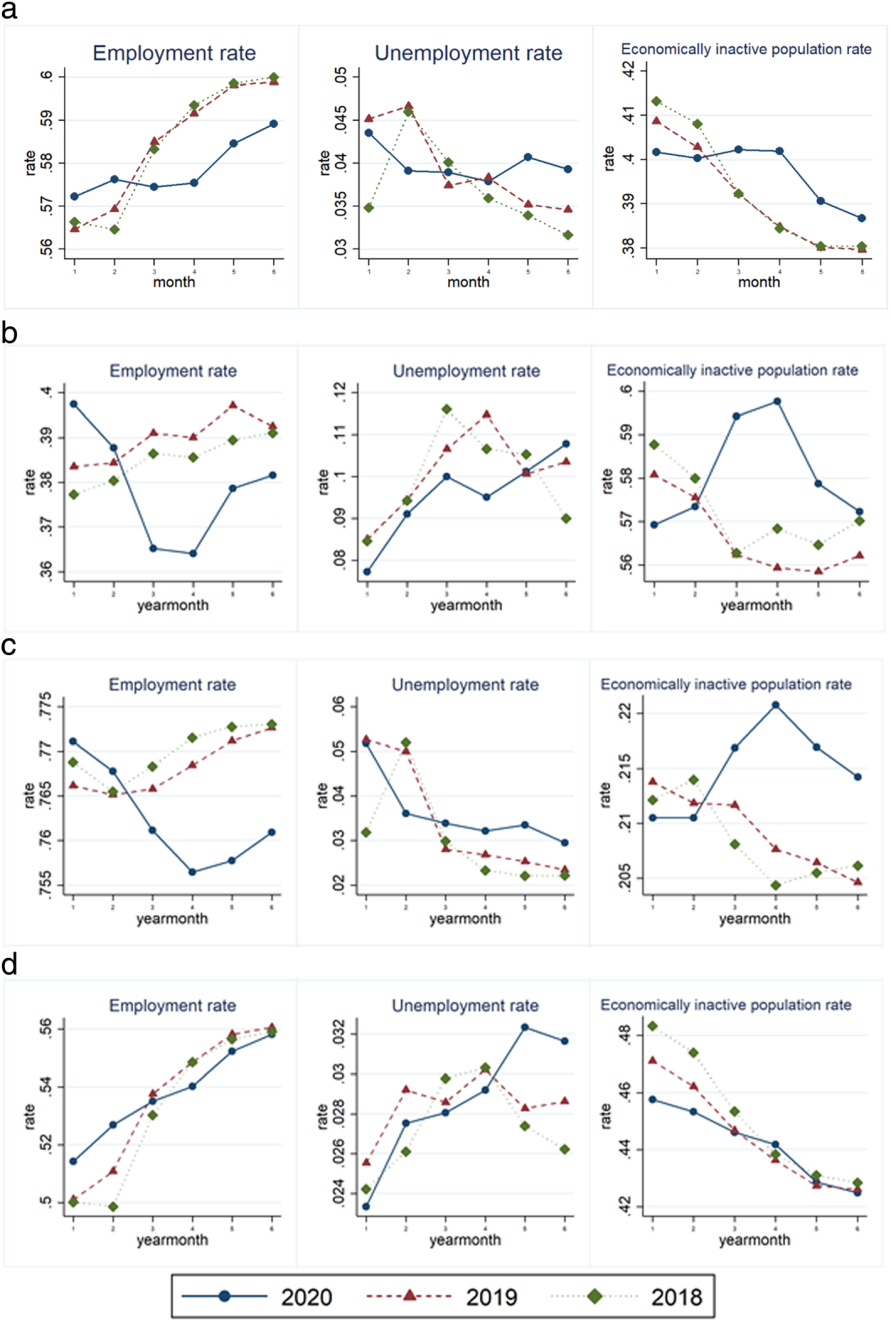
Employment, unemployment, and economic inactivity rates of all samples and for those aged 15–29 years. Panel A. All Samples. Panel B. Samples aged 15–29 years. Panel C. Samples aged 30–49 years. Panel D. Samples aged 50 over

Figure 3 shows the monthly trends for employment, unemployment, and economically inactive rates from January to June 2018, 2019, and 2020 in Korea. Panel A shows the labor market index for the entire sample, Panel B shows the same for young people aged between 15 and 29 years, Panel C shows the same for middle-aged people aged between 30 and 49 years, and Panel D shows the same for the elderly aged 50 and over.

Panel A mostly shows a similar trend between 2018 and 2019 that differs beginning in 2020. For instance, the employment rate dropped drastically in February 2020 when there were the maximum number of confirmed cases in Korea, decreasing by approximately 3.5% from January to April, and beginning to recover in May. The economically inactive population rate took a downturn beginning in May 2020.

Panel B also mostly shows a similar trend between 2018 and 2019 that begins to clearly differ in 2020. The unemployment rate decreased slightly in April and then started to increase in May, and the economically inactive population rates rapidly increased until April 2020. These data demonstrate the considerable impact of COVID-19 on the labor market for young people, who largely accounted for the increase in the economically inactive population rates.

In panel C, the employment rate continued in the downward trend until April and then turned upward from May. This trend is similar to that of the young people’s employment rate in 2020. On the contrary, the unemployment rate of the middle-aged population continued to decrease.

Panel D shows a different trend from panel B and C. The employment and unemployment rates continued to increase. The proportion of the economically inactive population continued to decrease.

### Empirical model

For this study, we used logistic regression to divide the population by youth and non-youths and estimated the correlations with the state of economic activity before and after COVID-19. Logistic regression is a typical nonlinear model for analysis commonly used when the dependent variables are not continuous but are discrete and categorical. The dependent variable used in this study is a binary variable with the value of 1 if an individual is economically inactive and 0 if the person is unemployed.

$$ {y}_{it}=\left\{\begin{array}{c}1, if{z}_{it}>0\\ {}0, if{z}_{it}\le 0\end{array}\right. $$

$$ where\ {z}_{it}={\beta}_0+{\beta}_1{Young}_i+{\beta}_2{Post}_t+{\beta}_3\left({Young}_i\times {Post}_t\right)+\sum \limits_{k=4}^K{\beta}_k{x}_{kit}+{u}_{it}(1) $$

In Eq. (1), i refers to the unemployed or economically inactive individual and t refers to time. *Young*_*i*_ is a dummy variable with the value of 1 if the individual i was between 15 and 29 years old. *Post*_*t*_ is a dummy variable with the value of 1 if the data observation time t was between January and June 2020. The interaction term is a variable to test the hypothesis that the young was more affected in the labor market by the COVID-19 outbreak. The control variables were gender, area of residence, year, previous job status, and the industry of the previous job. Here, *z*_*i*_ is presumed to be the sum of the error term and the linear combination of explanatory variables including constant terms. For logit regression, the logistic function of the error term was used as the probability function [28]:

$$ P\left({y}_{it}=1|X\right)=P\left({z}_{it}>0|X\right)=\frac{\mathit{\exp}\left({X}^{\prime }{\beta}_k\right)}{1+\mathit{\exp}\left({X}^{\prime }{\beta}_k\right)}(2) $$

Moreover, multinomial logit regression verified the correlations with the reasons for economic inactivity, by dividing the young people based on before and after the outbreak. The dependent variable is a multinomial variable, and include: “discouraged about job search,” “childcare, housework, and studying at an institution,” “mental/physical disabilities, others and non-response,” and “unemployment.” Here, unemployment served as the baseline outcome. Variables used in the analysis, including the control variables, were equivalent to those used in the logistic regression.

Ai and Norton (2003) highlighted the issue of interaction effects in nonlinear models [29]. To resolve this, Buis (2010) used the odds ratio and the relative risk ratio (RRR) to interpret a multiplier effect in the baseline effects of interaction terms [30]. We used Buis (2010)‘s method to interpret the interaction effect in this study [30].

### Estimation results

Table  4 illustrates the analysis of the effects of COVID-19 on economic activity. Table 4 tested the hypothesis that the COVID-19 outbreak had a differential effect on the unemployment and economic inactivity of young people versus that of the middle-aged or older group. To this end, we used the binary dependent variable representing unemployment or economic inactivity, and used the interaction term that combined with the dummy variables that represented the young people and post-COVID-19.

In Table  4, the odds ratio of the young variable showed a significant positive direction. Compared with the middle-aged or older group, the probability of being economically inactive in young people increased by 34.6%. The variable representing pre- and post-COVID-19 showed that the probability of being economically inactive increased by 3.1% after the outbreak compared with pre-COVID-19. For the interaction terms of young and COVID-19 outbreak, the main variables of interest in this study, young people × post-COVID-19 showed a significant positive direction: The probability that young people would become economically inactive after the COVID-19 increased by 53% compared with the middle-aged or older group before COVID-19.

**Table 4:** Results of logit regression (entire period of 2018–2020)

	Period: Jan.-Jun. 2018; Jan.-Jun. 2019; Jan.-Jun. 2020		
Variable		Odds ratio	**Robust S.E.**
Personal Characteristics	Women	1.889***	0.0000
	Region	1.126***	0.0000
	Middle School	1.009***	0.0000
	High School	0.571***	0.0000
	Two-year College	0.273***	0.0000
	Four-year College	0.275***	0.0000
	Master’s or Higher	0.401***	0.0000
	Married	2.701***	−0.0001
	Widowed	1.434***	0.0000
Industry	Agri., Forestry & Fishery	0.445***	0.0000
	Mfg. & Construction	0.0669***	0.0000
	Wholesale, Retail & Transport	0.110***	0.0000
	Lodging & Foodservice	0.139***	0.0000
	ICT & Sci. Tech.	0.0862***	0.0000
	Financial Services, Real Estate & Facility Mgt.	0.0764***	0.0000
	Public Admin. & Associations	0.108***	0.0000
	Healthcare	0.0709***	0.0000
	Arts	0.130***	0.0000
	Foreign	0.608***	0.0005
Treatment group	Young (ages 15–29)	1.346***	0.0000
Time	Post COVID-19	1.031***	0.0000
Interaction term	Young X Post COVID-19	1.153***	0.0000
time-trend		1.000***	0.0000
Constant		0***	0
Observations		317,233,504	

Note: Weights were used in the analysis

Note. Statistically significant at the significance level of ***p<0.01, **p<0.05, *p<0.1

Table 5 shows the results of the robustness check for the analysis results in Table 4. From previous basic statistics, the trend of the young people economically inactive population was not constant. The analysis period was limited and analyzed again. The first part of the table shows the results of limiting the time before COVID-19 to 2019, and the second part of the table shows the results of the analysis by limiting the time before COVID-19 to 2018. The two analysis results are confirmed to be similar to the results in Table 4. The overall analysis results are presented in Appendix 1 and Appendix 2.

**Table 5:** Robustness check, differential time trend

**Period: Jan.-Jun. 2019; Jan.-Jun. 2020**			
**Variable**		**Odds ratio**	**Robust S.E.**
Treatment group	Young (ages 15–29)	1.281***	0.0000
Time	Post COVID-19	1.062***	0.0000
Interaction term	Young X Post COVID-19	1.136***	0.0000
Observations		168,409,455,772	
**Period: Jan.-Jun. 2018; Jan.-Jun. 2020**			
**Variable**		**Odds ratio**	**Robust S.E.**
Treatment group	Young (ages 15–29)	1.343***	0.0000
Time	Post COVID-19	1.092***	0.0000
Interaction term	Young X Post COVID-19	1.132***	0.0000
Observations		168,202,783,240	

Note. Statistically significant at the significance level of ***p<0.01, **p<0.05, *p<0.1

Table 6 shows the result of analyzing the reasons for economic inactivity. In other words, it shows the result of multinomial logit regression with reasons for economic inactivity as the dependent variables (“discouraged about job search,” etc.). The period of analysis and control variables were equivalent to that of Table 4.

**Table 6:** Results of multinomial logit regression (entire period of 2018–2020)

			Period: 2018.01 ~ 06, 2019.01 ~ 06, 2020.01 ~ 06				
		a. Discouraged about job search		b. Housework, and studying at an institution		c. Others	
Variable		RRR	Robust S.E.	RRR	Robust S.E.	RRR	Robust S.E.
Treatment group	Young	0.932***	0.0000	1.123***	−0.0001	1.768***	− 0.0001
Time	Post Covid19	1.046***	0.0000	1.530***	−0.0001	1.015***	0.0000
Interaction term	Young X post	1.231***	−0.0001	1.193***	−0.0001	1.109***	0.0000
Observations		317,233,504		317,233,504		317,233,504	

Note. Statistically significant at the significance level of ***p<0.01, **p<0.05, *p<0.1

Compared to the middle-aged or older groups, the probability of being economically inactive for young people decreased by 6.8% due to abandonment of job search. However, after COVID-19, the probability of giving up finding a job increases. Since the outbreak of COVID-19, the probability of young people becoming economically inactive because they felt discouraged about their job search increased by 23.1% compared with that of the middle-aged or older group, reflecting that the post-COVID-19 search for work has been more difficult for young people than for other age groups.

When the reason for economic inactivity was childcare, housework, or studying at an institution, the impact was greater among young people. Moreover, as facilities like schools and daycare centers remained closed for social distancing, the probability of being economic inactivity for post-COVID-19 increased by 19.3%. For mental/physical disabilities, others, and non-response, the RRRs for young people were higher than that for the middle-aged. The overall analysis results are presented in Appendix 3.

Table 7 shows the result of the analysis on employability. As a result of the analysis, the probability that young people positivity towards finding jobs increased by 15.3% compared to middle-aged or older aged people. The probability that people positively believed in their employability decreases by 74.6% after the outbreak of COVID-19. Among them, the probability that young people positively believed in the possibility of employment after COVID-19 decreased even more.

**Table 7:** Employability analysis

	Period: Jan.-Jun. 2018; Jan.-Jun. 2019; Jan.-Jun. 2020		
Variable		Odds ratio	Robust S.E.
Personal Characteristics	Women	0.663***	0.0000
	Region	0.958***	0.0000
	Middle School	0.940***	0.0001
	High School	0.728***	0.0000
	Two-year College	0.679***	0.0000
	Four-year College	0.768***	0.0000
	Master’s or Higher	0.422***	0.0000
	Married	0.647***	−0.0001
	Widowed	0.551***	0.0000
Industry	Agri., Forestry & Fishery	1.435***	0.0000
	Mfg. & Construction	1.070***	0.0000
	Wholesale, Retail & Transport	0.910***	0.0000
	Lodging & Foodservice	0.924***	0.0000
	ICT & Sci. Tech.	1.030***	0.0002
	Financial Services, Real Estate & Facility Mgt.	1.104***	0.0001
	Public Admin. & Associations	1.243***	0.0001
	Healthcare	1.027***	0.0003
	Arts	1.306***	0.0002
	Foreign	0.570***	0.0001
Treatment group	Young (ages 15–29)	1.153***	0.0000
Time	Post COVID-19	0.254***	0.0000
Interaction term	Young X Post COVID-19	0.543***	0.0000
time-trend		1.001***	0.0000
Constant		0***	0
Observations		31,410,004,412	

Note. Statistically significant at the significance level of ***p<0.01, **p<0.05, *p<0.1

## Discussion

The results of this study can be summarized in three points. First, the employment rate in Korea declined after the outbreak of COVID-19, and most of the unemployed became a part of the economically inactive population. Second, young people were more likely than the other age groups to become economically inactive. Third, young people were more likely after the outbreak to become economically inactive because of disappointment about their job searches. Young people also reacted negatively to the possibility of employment in the future.

The COVID-19 pandemic negatively impacted the Korean labor market with its rapid spread in two ways. The first is due to the fact that many people who are infected with the disease remain asymptomatic, with Korean public health authorities identifying that approximately 30% of the confirmed cases in the country were asymptomatic [31]. Moreover, the source of infection was unknown for 10% of the confirmed cases, which was possibly attributable in part to young people who could be regarded as undetected but fearful.

The second is social distancing and self-quarantine, which are some of the various quarantine measures implemented by the Korean government. In order to prevent the spread of COVID-19 in advance, contact between individuals was minimized in all parts of daily life, and self-quarantine was required in the case of contact with confirmed COVID-19 patients. Particularly, social distancing required drastic changes to not only public service facilities such as schools, but also to private workplaces. The changes mean changing the working environment to become far more contactless. The World Bank (2014) identified two ways pandemics affect economies, one of which is through behavioral changes triggered by the fear of contracting the disease [32]. Quarantine and social distancing are also examples of behavioral changes due to the fear of infection. Other examples of behavioral change include decreased participation in the labor market and the shutdown of workplaces to avoid physical contact. Analogous to Lee & Mckibbin (2004), the negative impacts of a pandemic on an economy originate from mental and behavioral contractions [33, 34].

In other words, the characteristics of COVID-19, such as infection without symptoms and the government’s preventive measures, such as social distancing, may reduce economic activities. This appears to be related to the increase of the economically inactive population and discouraged young job seekers, as shown in this study.

## Conclusion

Korea was acknowledged internationally for its exemplary handling of the COVID-19 crisis, but the economic recession caused by the disease affected the corporate behavior of personnel management, who reduced recruitment schemes, thereby resulting in negative implications for young people, who are particularly vulnerable within the Korean labor market. Young people in South Korea possess little career experience in the labor market and tend to be seeking work rather than working. Because economic activities are likely to shrink structurally during a pandemic, it is necessary to empirically determine the damage incurred by people who are vulnerable in the labor markets, such as the younger population which was the subject of this study.

This study empirically proved COVID-19’s adverse effect on young people in South Korea who although medically strong, are vulnerable in the labor market. The logit and multinomial logit regression were used according to the state of economic activity, and interaction terms were added to verify how the impact of COVID-19 varies among age group (young people, the middle-aged, or older people). The EAPS data used in this analysis were from January to June 2018, 2019, and 2020, in order to compare the numbers before and after the outbreak of COVID-19.

This study differs from prior studies in two ways. First, it empirically analyzed the labor market conditions before and after the outbreak of COVID-19 using massive micro-data, thereby determining how COVID-19 affected the labor market in Korea despite exemplary handling of the COVID-19 situation. Second, it analyzed the differential impact of COVID-19 by taking the different outcomes of various age groups. It empirically verified that the impact of COVID-19 varies depending on whether one is young or not, as each age group is exposed to different circumstances within the labor market. Our empirical results revealed reasons for economic inactivity, such as “discouragement about job search” and “childcare, housework, and studying at an institution.” Young people showed a significantly higher relative probability of becoming economically inactive or discouraged job seekers following the COVID-19 outbreak when compared to that of other age groups. This study also has one limitation. This study failed to analyze the causal effect. It simply presents the phenomenon that appears in the Korean labor market due to the outbreak of COVID-19 as a correlation analysis.

Workers who become unemployed in Korea must first be officially determined as being eligible for unemployment benefits at employment centers, which are supervised by the Ministry of Employment and Labor, in order to receive unemployment benefits under the employment insurance scheme. However, young people entering the labor market for the first time often work as part-timers at workplaces which are not covered by the employment insurance scheme, and are therefore more likely to experience the hopping path of employed-economically inactive-employed. Young people are increasingly missing out on employment opportunities during their job-seeking periods, and the increasing trend of young people becoming a part of the long-term economically inactive population has been aggravated after the COVID-19 outbreak. The fact that young people are more concentrated in the economically inactive population rather than in the unemployed pool compared to other age groups following the COVID-19 outbreak suggests that it is highly likely that young people will be placed in the blind spot of employment insurance, Korea’s major social safety net, compared to other age groups. To make matters worse, if the government were to set a goal of reducing unemployment in young people, it will mean that the increasing population of economically inactive young people will be excluded from policy focus, and additionally, that young people are highly likely to be placed in the blind spot of the government’s employment policy, in addition to the aforementioned blind spot of the social safety net.

The reason that economic inactivity has increased more among young people compared to other age groups after the COVID-19 outbreak, is additionally attributable to the sharp decline in labor demand for non-regular workers and for employees at small- and medium-sized enterprises (SMEs). Even though SMEs possess a lower ability to pay their employees, they had previously hired many young people at their early entry stage of the labor market. However, SMEs have become more reluctant in hiring young people who have no or very little work experience, following the COVID-19 outbreak, as they need to hire more essential workers to do face-to-face work at the company sites, not at homes. The Korean labor market is divided into the core and the peripheral sectors, with the core sector comprised mainly of large corporations with regular employees, and the peripheral sector comprised of SMEs with non-regular employees. Young people often gain work experience in the peripheral sector before moving onto the core sector [35, 36]. The core labor sector with higher wage-paying ability and relatively higher wages has actively promoted contactless telecommuting at home for quarantine purposes following the COVID-19 outbreak. On the other hand, the peripheral labor sector, where young people are concentrated - and which is characterized by lower wage-paying ability and relatively lower wages – requires mostly face-to-face work. Furthermore, with the peripheral labor market being hit harder and more unequally by the COVID-19 outbreak, it can be inferred that more employment opportunities for young people would have been lost in this sector.

Therefore, maintaining the conventional social safety net centered on fully employed individuals and the conventional employment policies centered on unemployment benefits will thereby be insufficient in preparing for the rapidly increasing population of economically inactive young people following the COVID-19 outbreak. More targeted and customized policies may be necessary in order to facilitate the school-to-work transition of young people who have experienced significant challenges due to the COVID-19 crisis.

## Data Availability

This study uses public-use data from the EAPS. All data can be found at https://mdis.kostat.go.kr/

## Appendix 1. Robustness check, differential time trend

**Table:**

	Period: Jan.-Jun. 2018; Jan.-Jun. 2020		
Variable		Odds ratio	Robust S.E.
Personal Characteristics	Women	1.953***	0.0000
	Region	1.042***	0.0000
	Middle School	1.040***	0.0000
	High School	0.621***	0.0000
	Two-year College	0.302***	0.0000
	Four-year College	0.314***	0.0000
	Master’s or Higher	0.403***	0.0000
	Married	2.278***	−0.0001
	Widowed	1.028***	0.0000
Industry	Agri., Forestry & Fishery	0.435***	0.0000
	Mfg. & Construction	0.0745***	0.0000
	Wholesale, Retail & Transport	0.116***	0.0000
	Lodging & Foodservice	0.144***	0.0000
	ICT & Sci. Tech.	0.0981***	0.0000
	Financial Services, Real Estate & Facility Mgt.	0.0843***	0.0000
	Public Admin. & Associations	0.137***	0.0000
	Healthcare	0.0933***	0.0000
	Arts	0.127***	0.0000
	Foreign	0.272***	0.0005
Treatment group	Young (ages 15–29)	1.281***	0.0000
Time	Post COVID-19	1.062***	0.0000
Interaction term	Young X Post COVID-19	1.136***	0.0000
time-trend		1.000***	0.0000
Constant		24.00***	0.0013
Observations		168,409,455,772	

Note. Statistically significant at the significance level of ****p*<0.01, ***p*<0.05, **p*<0.1

## Appendix 2. Robustness check, differential time trend

**Table:**

	Period: Jan.-Jun. 2019; Jan.-Jun. 2020		
Variable		Odds ratio	Robust S.E.
Personal Characteristics	Women	1.933***	0.0000
	Region	1.170***	0.0000
	Middle School	1.151***	0.0000
	High School	0.615***	0.0000
	Two-year College	0.281***	0.0000
	Four-year College	0.281***	0.0000
	Master’s or Higher	0.412***	0.0000
	Married	2.504***	−0.0001
	Widowed	1.034***	0.0000
Industry	Agri., Forestry & Fishery	0.452***	0.0000
	Mfg. & Construction	0.0797***	0.0000
	Wholesale, Retail & Transport	0.129***	0.0000
	Lodging & Foodservice	0.173***	0.0000
	ICT & Sci. Tech.	0.0991***	0.0000
	Financial Services, Real Estate & Facility Mgt.	0.0941***	0.0000
	Public Admin. & Associations	0.149***	0.0000
	Healthcare	0.110***	0.0000
	Arts	0.140***	0.0000
	Foreign	0.391***	0.0005
Treatment group	Young (ages 15–29)	1.343***	0.0000
Time	Post COVID-19	1.092***	0.0000
Interaction term	Young X Post COVID-19	1.132***	0.0000
time-trend		1.000***	0.0000
Constant		21.23***	0.00114
Observations		168,202,783,240	

Note. Statistically significant at the significance level of ****p*<0.01, ***p*<0.05, **p*<0.1

## Appendix 3. Results of multinomial logit regression (entire period of 2018–2020)

A. Discouraged about job search

**Table:**

	Period: Jan.-Jun. 2018; Jan.-Jun. 2020		
Variable		RRR	Robust S.E.
Personal Characteristics	Women	1.094***	0.0000
	Region	1.135***	0.0000
	Middle School	0.723***	0.0000
	High School	0.936***	0.0000
	Two-year College	0.868***	0.0000
	Four-year College	0.912***	0.0000
	Master’s or Higher	0.816***	0.0000
	Married	0.968***	−0.0000
	Widowed	0.786***	0.0000
Industry	Agri., Forestry & Fishery	0.678***	0.0000
	Mfg. & Construction	0.250***	0.0000
	Wholesale, Retail & Transport	0.245***	0.0000
	Lodging & Foodservice	0.313***	0.0000
	ICT & Sci. Tech.	0.211***	0.0000
	Financial Services, Real Estate & Facility Mgt.	0.232***	0.0000
	Public Admin. & Associations	0.307***	0.0000
	Healthcare	0.204***	0.0000
	Arts	0.252***	0.0001
	Foreign	0.452***	0.0000
Treatment group	Young (ages 15–29)	0.00150***	0.0000
Time	Post COVID-19	0.938***	0.0000
Interaction term	Young X Post COVID-19	1.055***	0.0000
time-trend		1.190***	0.0000
Constant		1.000***	0.0000
Observations		251,732,418,319	

B. Housework, and studying at an institution

**Table:**

	Period: Jan.-Jun. 2018; Jan.-Jun. 2020		
Variable		RRR	Robust S.E.
Personal Characteristics	Women	2.117***	0.0000
	Region	1.125***	0.0000
	Middle School	0.936***	0.0000
	High School	1.435***	0.0001
	Two-year College	1.289***	0.0001
	Four-year College	1.146***	0.0001
	Master’s or Higher	1.309***	0.0002
	Married	1.583***	−0.0000
	Widowed	0.885***	0.0000
Industry	Agri., Forestry & Fishery	0.975***	0.0001
	Mfg. & Construction	0.236***	0.0000
	Wholesale, Retail & Transport	0.302***	0.0000
	Lodging & Foodservice	0.410***	0.0000
	ICT & Sci. Tech.	0.135***	0.0000
	Financial Services, Real Estate & Facility Mgt.	0.214***	0.0000
	Public Admin. & Associations	0.332***	0.0000
	Healthcare	0.183***	0.0000
	Arts	0.295***	0.0004
	Foreign	1.444***	0.0000
Treatment group	Young (ages 15–29)	0.002***	0.0000
Time	Post COVID-19	1.104***	0.0001
Interaction term	Young X Post COVID-19	1.507***	0.0000
time-trend		1.189***	0.0000
Constant		1.000***	0.0000
Observations		251,732,418,319	

C. Others

**Table:**

	Period: Jan.-Jun. 2018; Jan.-Jun. 2020		
Variable		RRR	Robust S.E.
Personal Characteristics	Women	1.094***	0.0000
	Region	1.135***	0.0000
	Middle School	0.723***	0.0000
	High School	0.936***	0.0000
	Two-year College	0.868***	0.0000
	Four-year College	0.912***	0.0000
	Master’s or Higher	0.816***	0.0000
	Married	0.968***	−0.0001
	Widowed	0.786***	0.0000
Industry	Agri., Forestry & Fishery	0.678***	0.0000
	Mfg. & Construction	0.250***	0.0000
	Wholesale, Retail & Transport	0.245***	0.0000
	Lodging & Foodservice	0.313***	0.0000
	ICT & Sci. Tech.	0.211***	0.0000
	Financial Services, Real Estate & Facility Mgt.	0.232***	0.0000
	Public Admin. & Associations	0.307***	0.0000
	Healthcare	0.204***	0.0000
	Arts	0.252***	0.0000
	Foreign	0.452***	0.0005
Treatment group	Young (ages 15–29)	0.00150***	0.0000
Time	Post COVID-19	0.938***	0.0000
Interaction term	Young X Post COVID-19	1.055***	0.0000
time-trend		1.000***	1.190***
Constant		24.00***	1.000***
Observations		251,732,418,319	

Note. Statistically significant at the significance level of ****p*<0.01, ***p*<0.05, **p*<0.1

## Abbreviations

COVID-19: SARS-CoV-2 virus
EAPS: Economically Active Population Survey
GDP: Gross Domestic Product
ILO: International Labour Organization
KCDC: Korea Central Disease Control Headquarters
MERS: Middle East Respiratory Syndrome
SARS: Severe Acute Respiratory Syndrome Coronavirus 2
SME: Small- and Medium-sized Enterprises
WHO: World Health Organization
